# Invasive Group B Streptococcal Infection in Infants, Malawi

**DOI:** 10.3201/eid1302.060680

**Published:** 2007-02

**Authors:** Katherine J. Gray, Sally L. Bennett, Neil French, Amos J. Phiri, Stephen M. Graham

**Affiliations:** * Malawi-Liverpool–Wellcome Trust Programme of Clinical Tropical Research, Blantyre, Malawi; † College of Medicine, Blantyre, Malawi

**Keywords:** Group B Streptococcus, Africa, epidemiology, serotype, neonatal disease, research

## Abstract

Group B streptococci (GBS) are a recently identified cause of neonatal sepsis in Malawi. In Queen Elizabeth Central Hospital, Blantyre, Malawi, during May 2004–June 2005, GBS were isolated from routine blood and cerebrospinal fluid cultures from 57 infants. The incidence of early (EOD) and late onset (LOD) invasive GBS disease was 0.92 and 0.89 cases per 1,000 live births, respectively. Sepsis (52%) was the most common manifestation of EOD; meningitis (43%) and sepsis (36%) were the principal manifestations of LOD. The case-fatality rate was 33% overall (38% EOD, 29% LOD). Serotypes Ia and III were responsible for 77% of disease. All isolates were susceptible to penicillin, but 21% were resistant to erythromycin. The rate and manifestations of neonatal GBS disease in Malawi are similar to those in industrialized countries, but the case-fatality rate is higher than in industrialized countries. Effective locally relevant prevention strategies are needed.

## Abstract 2

Incidence and serotype distribution of disease in Malawi are similar to those reported from industrialized countries, but case-fatality rate is high.

Group B streptococcus (GBS) has been a leading cause of neonatal illness and death in many parts of the world, especially industrialized countries, for several decades (*1**–**5*). In contrast, until recently GBS was infrequently reported in the developing world. A World Health Organization multicenter study of the bacterial etiology of serious infections in young infants of <3 months of age reported in 1999 that the “virtual absence of GBS was striking” (*6*). Yet the prevalence of maternal carriage of GBS in developing countries, including populations in tropical Africa, is similar to that identified in populations in the United States (*7**–**9*). Recent studies from Kenya (*10**–**12*), South Africa (*13**,**14*), Zimbabwe (*15*)*,* and Malawi (*16*) suggest that GBS is emerging as an important cause of neonatal sepsis in Africa. The largest of these studies reported that 136 of 801 bacterial isolates from 784 Malawian neonates were GBS, which makes it the most common cause of sepsis among neonates admitted to Queen Elizabeth Central Hospital (QECH) in Blantyre (*16*).

Prevention strategies such as chemoprophylaxis are available for neonatal GBS but are difficult to apply in a resource-limited setting (*4**,**5*). Vaccination is an attractive option in this setting, and vaccines consisting of GBS capsular polysaccharide conjugated to a tetanus toxoid carrier protein have been under development (*17**–**20*). The vaccines are immunogenic in women but of unproven clinical benefit. Important information to support future preventive strategies includes estimate of rates of disease, timing of disease initial manifestations; and for vaccine development, description of serotype distribution in different populations (*5*). Therefore, we set out to further characterize GBS disease in Blantyre District in Malawi.

## Methods

### Study Setting

The study was conducted during 14 months from May 1, 2004, to June 30, 2005, at QECH in Blantyre District. This district has the largest urban population in Malawi, and much of the population lives in impoverished townships. The predicted midyear population in 2005 was 1,070,173 (www.nso.malawi.net). This estimate is based on projections from the 1998 national census. QECH is an urban district hospital, which takes direct admissions and referrals from surrounding district health centers. It is the only major hospital providing free care in Blantyre. Birth and death statistics for Blantyre for the study period were obtained directly from QECH and the Blantyre District Health Office.

### Study Population

Neonates (birth to 6 days of age) are normally admitted directly to the neonatal nursery from the labor ward or postnatal wards. Neonates may also be referred from surrounding health centers in Blantyre District if problems occur immediately after delivery. Young infants from birth to 6 months of age (including those from birth to 90 days of age) who were discharged well after delivery at QECH or in peripheral health centers but in whom symptoms suggestive of sepsis subsequently developed are normally admitted to the pediatric ward. What proportion of infants with sepsis in Blantyre is seen at healthcare facilities is not known.

Guidelines exist for the investigation of sick children. Cerebrospinal fluid (CSF) should be taken from all children with suspected meningitis as well as blood cultures, when there is evidence of sepsis (temperature >38°C) but no signs to suggest localized disease. In practice this means most neonates with nonspecific signs will have both blood and CSF cultures taken before empirical antimicrobial agents are administered. Infants >1 month of age will only have a blood culture taken if no clear focus of infection, e.g., pneumonia, is evident. Guidelines exist for the use of intrapartum antimicrobial agents in febrile mothers with suspected chorioamnionitis. If prolonged rupture of membranes occurs and the neonate is admitted to the neonatal nursery, antimicrobial agents are given empirically to the infant. No record or audit information is available to assess adherence to the guidelines.

Culture of GBS from a blood or CSF sample from a QECH pediatric inpatient <90 days of age was the entry point to the study. Positive samples initiated a visit to the patient and the collection of clinical and, later, outcome data on the child. If a child had died with a positive GBS culture, the death was attributed to GBS. No autopsy results were available.

Most births take place at health facilities. Eighty-three percent of women who live in an urban setting will deliver at a health clinic or hospital (Malawi Demographic and Health Survey preliminary report; www.nso.malawi.net). HIV prevalence in mothers delivering at QECH was 30.2% from 2000 to 2004 (*21*)

### Data Collection

Information on admissions to the neonatal nursery and pediatric ward and the number of blood cultures taken was obtained from ward admission books and laboratory records. The clinical notes of patients from whom GBS was isolated were reviewed. When no notes were available (e.g., because of death or discharge of the child before GBS was identified), the ward admission, ward round, and books containing information about patients who died on the ward were used to provide data. Information collected included date of birth, age, sex, district of residence, birthweight, and gestational age at birth (defined by maternal dates). If a child was born before 37 weeks’ gestation or weighed <2.5 kg, he or she was classified as premature or of low birthweight (LBW), respectively. Age at onset of illness was used to classify the child’s condition as early onset disease (EOD, defined as disease starting from birth to 6 days after birth), or late onset disease (LOD, defined as 7–90 days inclusive after birth). Outcome in hospital was recorded as dead or alive at discharge. No attempt was made to actively follow up the patients after discharge.

### Clinical Definitions

Disease type was categorized by using the following criteria: 1) meningitis, pyogenic CSF from which GBS was grown; 2) probable meningitis, no GBS isolated from CSF but GBS isolated from blood and CSF findings consistent with meningitis; 3) sepsis, GBS isolated from blood with no clinical evidence of pneumonia, i.e., no increased respiratory rate or chest retraction; 4) pneumonia, GBS isolated from blood and definite clinical evidence of pneumonia, i.e., increased respiratory rate or chest retraction; 5) unknown, GBS isolated from blood but insufficient information to clinically categorize patient. The study was approved by the College of Medicine Research and Ethics Committee of the University of Malawi.

### Laboratory Methods

Blood cultures are processed with a commercial blood culturing system (BacT Alert, biomérieux, Lyons, France). CSF is processed by using standard methods. Positive blood and CSF isolates are cultured on standard media by using routine techniques. GBS was identified by its β-hemolysis on blood agar (α-hemolytic and nonhemolytic streptococci were not evaluated); negative catalase reactions and serogrouping were conducted by using a latex agglutination test (Pro-Lab Diagnostics, Wirral, UK). Serotyping of the GBS isolates was performed with a commercial serotyping kit according to the manufacturer’s instructions (Statens Serum Institut, Copenhagen, Denmark).

Disk-diffusion antimicrobial susceptibility testing was performed according to the British Society for Antimicrobial Chemotherapy guidelines on Isosensitest agar (Oxoid Ltd, Basingstoke, UK) supplemented with 5% sheep blood media (*22*). Antimicrobial agents tested included penicillin, tetracycline, erythromycin, chloramphenicol, and ceftriaxone. All laboratory procedures were internally quality controlled. The laboratory is enrolled in the United Kingdom National External Quality Assessment Service for Microbiology.

## Results

### Clinical Characteristics

GBS was isolated from 57 infants in the 14-month study period; of these, 41 isolates were from blood culture only, 7 from both blood and CSF, and 9 from CSF alone. With respect to the blood cultures, 3,159 infants were admitted to the neonatal nursery during the study period; blood cultures were drawn from 681 (22%) of these patients, and 117 (17%) grew a clinically relevant isolate; 26 (22%) of these isolates were GBS. There were 4,297 children admitted to the pediatric ward; blood cultures were drawn from 1,652 (38%) of these patients, and 173 (10%) grew a clinically relevant isolate; 22 (13%) of these isolates were GBS. Admission numbers and blood cultures could not be accurately analyzed by age for the patient for the pediatric ward. Of the 57 patients, 19 died, 35 were discharged, and the outcome of 3 patients was not ascertained. The overall case-fatality rate was 33%. The Table contains a summary of the major clinical findings.

Seven (16%) of 45 infants with known gestational age were preterm, and 10 (20%) of 51 infants with known birthweight had LBW. Whether disease was early or late onset was not associated with these variables. Meningitis was more common among infants with LOD than those with EOD (Table), but the difference did not reach statistical significance (χ^2^ = 3.4, p = 0.07).

**Table:** Characteristics of 57 case-patients with group B streptococcal infection overall and in relation to capsular serotype

		Serotype					
Clinical features	Total (%)	Ia (n = 12)	Ib (n = 3)	Ll (n = 4)	Lll (n = 32)	V (n = 1)	Unknown (n = 5)
Early onset disease*	29	7	2	3	14	1	2
Male sex†	15 (52)‡	6	2	2	4	0	1
Meningitis	5 (17)	1	1	1	2	0	0
Probable meningitis	4 (14)	1	0	0	3	0	0
Sepsis	15 (52)	5	1	1	8	0	0
Pneumonia	0 (0)	–	–	–	–	–	–
Undefined	5 (17)	0	0	1	1	1	2
Low birthweight§¶	5 (17)	2	1	0	2	0	0
Premature#**	3 (10)	2	0	0	1	0	0
Late onset disease††	28	5	1	1	18	0	3
Male sex‡‡	13 (46)	2	1	1	9	0	0
Meningitis	11 (39)	2	0	0	8	0	1
Probable meningitis	1 (4)	0	0	0	1	0	0
Sepsis	10 (36)	1	1	0	8	0	0
Pneumonia	3 (11)	1	0	1	1	0	0
Undefined	3 (11)	1	0	0	0	0	2
Low birthweight§§	5 (18)	2	0	1	1	0	1
Premature¶¶	4 (14)	2	0	1	1	0	0
Case fatality‡							
Early onset disease	11 (38)	3	0	0	8	0	0
Late onset disease	8 (29)	3	0	0	4	0	1
Unknown	3 (5)	0	0	1	1	0	1
Sensitivity, %							
Penicillin	100	100	100	100	100	100	100
Erythromycin	79	90	100	67	78	0	78
Tetracycline	4	17	0	0	0	0	11
Ceftriaxone	100	100	100	100	100	100	100
Chloramphenicol	81	90	100	100	78	0	77

*Illness onset between birth and day 6. †Missing information on 3 cases. ‡Expressed as a proportion of total early or late onset cases. §Weight at birth <2.5 kg. ¶Missing information on 2 cases. #Delivery before 37 weeks of gestation. **Missing information on 6 cases. ††Illness onset ≥7 d after delivery. ‡‡Missing information on 2 cases. §§Missing information on 4 cases. ¶¶Missing information on 6 cases.

Of the isolates, 29 (51%) were from infants with EOD, and the median age of patients with initial symptoms was 1 day. The case-fatality rate was 38% for EOD. Twenty-eight isolates (49%) were from infants with LOD. The median age of LOD was 14 days (range 7–42 days), and the case-fatality rate was 29%.

### Serotypes

Of the 57 patients in whom GBS was identified, 52 had isolates available for serotyping. GBS were isolated from both blood and CSF in 7 cases, but both isolates were available for typing in only 4 cases; in all of these cases, the serotypes were the same. Thus, only 1 isolate per infant was included in the analysis. No GBS isolates were nontypeable. Serotype III (56%) and serotype Ia (21%) were the most frequently identified serotypes; they constituted 77% of both EOD and LOD (Figure).

**Figure:**
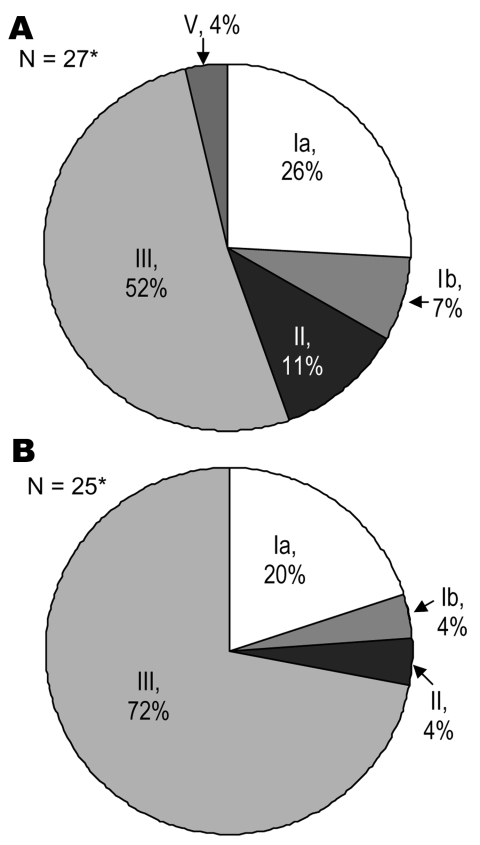
Pie chart showing serotype distribution of group B streptococcus isolates from infants with early (A) or late onset (B) disease. *Two isolates from early onset disease and 3 from late onset disease were not available for typing.

Disease manifestations by serotype are shown in the Table. No discernible differences were found in EOD or LOD, clinical manifestations, or outcome by serotype. Of the 51 infants for whom a birthweight was recorded, serotype Ia caused more disease among LBW babies than among those of normal birthweight, but the trend was not significant (40% vs. 17%, respectively, χ^2^ = 3.1, p = 0.08). Disease due to serotype III was less common in those of LBW (30% vs. 68%, respectively, χ^2^ = 4.3, p = 0.04).

All GBS isolates were susceptible to penicillin, and all but 2 isolates were resistant to tetracycline (Table). Serotype and antimicrobial susceptibility were not statistically associated.

### Incidence Rate Estimates

During the study period, May 1, 2004–June 30, 2005, a total of 31,458 live births were recorded in Blantyre District; a birth rate of 25.2/1,000 population. Of these births, 12,064 took place in QECH and 19,394 took place in district health centers. Therefore, the overall GBS disease incidence was 1.8/1,000 live births. The incidence of EOD was 0.92/1,000 live births, and the incidence of LOD was 0.89/1,000 live births. During the study period, 711 neonatal deaths (23% of all admissions) occurred in the neonatal nursery. A further 353 deaths (8% of all admissions) occurred in the pediatric ward, but these deaths could not be analyzed by age. GBS was implicated as a cause of death in 11 (2%) of the deaths in the neonatal nursery and in 8 (2%) of all the deaths in the pediatric ward.

## Discussion

This study adds to the growing evidence that GBS is an important cause of infectious neonatal illness and death in Africa. The incidence and outcome of disease support a more active approach for its prevention.

These results provide a benchmark for future studies with what we believe to be reasonable minimum estimates of disease incidence, despite measurement limitations in both our denominator and numerator figures. The recorded number of live births during the study period for Blantyre District is almost certainly an underestimate of the actual number. Our calculated birth rate of 25/1,000 population is low for an African urban population. A recent household demographic survey estimated the birth rate in urban Malawi at 37/1,000 (www.nso.malawi.net); thus, our live birth numbers may be underrecorded by as much as one third.

Set against this background, case-ascertainment of GBS was also suboptimal. Surveillance for GBS was passive. Only 1 in 5 infants admitted to the neonatal nursery and 2 in 5 admitted to the pediatric ward had a blood culture performed as part of the investigation of their illness. Although guidelines for assessing sick neonates exist, no audit of their implementation has been undertaken in the hospital, and shortages of syringes, needles, blood tubes, and staff are commonplace. The relatively low numbers of EOD to LOD and the high number of deaths may also be in part explained by selective sampling of the sicker children, rather than a fundamental difference in disease pathology in Malawi.

What proportion of sick neonates was seen in QECH and how many died before they received any form of healthcare are unclear. Using data from the household demographic survey (a birth rate of 37/1,000 population and a reported neonatal death rate of 27/1,000 births [www.nso.malawi.net]) and the projected population size for Blantyre (www.nso.malawi.net/data_on_line/demography/projections/pop/bt_rural htm and bt_city.htm), we would have expected ≈1,250 neonatal deaths in Blantyre during the study period. The 711 recorded deaths in the neonatal nursery and a proportion of the 353 deaths on the pediatric ward suggest that most neonatal deaths in Blantyre occur in QECH, but a sizeable proportion do not. We believe our results are likely to underestimate rates of GBS disease with the extent of lack of case recognition being greater than the underreporting of births.

The overall rate of GBS disease in Blantyre is higher than the overall rates of 0.6–0.9/1,000 live births reported from Western Europe (*3**,**23**,**24*). However, the rate of EOD is lower than that documented in the United States and Australia before the use of intrapartum prophylaxis, 1.7–2.0/1,000 live births, (*1**,**2*). Little information is available about rates of invasive disease in Africa for comparison. A study from the principal public-funded hospital in Johannesburg, South Africa, reported an EOD rate of 2.06/1,000 live births (*14*). That study used similar methods to our own for the rate calculations, although the calculated crude birth rate from the figures reported (≈18/1,000 population) suggests underreporting of births for the denominator and overall rates that may be similar to those in Blantyre. Another study from Johannesburg reported an EOD incidence rate of 1.16/1,000 live births (*13*) although the sociodemographic background of the population under study here is less clear. In a rural setting in East Africa, GBS bacteremia occurred at a rate of 0.66/1,000 births in neonates (*10*), which suggests that the extent of disease is greater in urban or southern Africa at this time.

The rates of EOD and LOD in this study were similar. In other settings, EOD is much more frequent than LOD when prophylaxis is not available. Our findings may in part be explained by selective sampling, but other factors may have also contributed. Some cases of EOD may have been prevented by empirical administration of antimicrobial agents, in keeping with the guidelines for chorioamnionitis and prolonged rupture of membranes, although we have no information as to the extent of this practice. Another possibility is that some of our LOD was in fact EOD because the patients had symptoms of illness for some time before seeking healthcare.

We found serotypes III and Ia to be the predominant serotypes, comprising 77% of cases; serotypes II, Ib, and V constituted the rest. This breakdown is similar to that in the single other report from Africa to date that assessed serotypes. That study, from South Africa, showed that in infants with EOD serotype III isolates caused 49.2% of disease and, together with serotype Ia isolates, caused 78.9% of disease (*14*). Studies from the industrialized world, in Finland (*25*) and Sweden (*26*), found a similar predominance of III and Ia. We found only 1 case of serotype V disease in contrast with findings from more recent studies from England (*3*), Sweden (*27*), and the United States (*28*), where serotype V is increasingly recognized as a cause of invasive disease. Serotype V was the predominant serotype, however, in a large Gambian study of maternal colonization (*8*) and was frequently identified in a similar Zimbabwean study (*29*). Neonatal disease was uncommon in the Gambian study, which suggests that factors other than bacterial serotype are required for disease to occur.

We found the rate of LOD, 0.89/1,000 live births, was slightly less than that of 1/1,000 live births reported in the South African study (*14*), although serotypes III and Ia were similarly responsible for most cases. We did not, however, define an association between serotype and timing of disease. These findings differ from reports from the industrialized world and from South Africa, where serotype III is clearly associated with LOD. This finding may also be a consequence of a case-finding bias with the youngest and sickest being more selectively investigated. The median age of patients with LOD in our study was 14 days; only 1 case occurred after the child was 28 days of age. This finding could be because hospitalized infants >28 days of age are less likely to have a blood culture taken if they have localized signs of sepsis, e.g., pneumonia. A more systematic and definitive approach to sampling will be required to further assess this finding.

Disease manifestations were similar to those in other studies, apart from a higher proportion of EOD (31%) manifesting as meningitis. Other studies have reported 6%–10% of EOD as meningitis (*2**,**3*). The high rate could be explained by preferential sampling of the sickest infants in circumstances of limited resources. We found that reliably differentiating sepsis from pneumonia was problematic, again, as a result of the lack of investigative facilities; thus, we may have underdiagnosed cases of pneumonia.

The case-fatality rate in this case-series resembles that seen in the United States in the 1970s, when the case-fatality rate was >50% (*30**,**31*). Our case-fatality rate is much higher than that more recently recorded in Europe (8%–9%) (*3**,**25**,**32*), the United States (4%–6%) (*2*), or South Africa (19.8% for EOD and 13.6% for LOD) (*14*). This finding likely reflects the difficulties of managing these infants with limited resources, lack of intensive care facilities, and late seeking of healthcare for some infants, and possibly coexistent illness such as HIV.

We do not have any information on HIV status of mothers or children in our study. Speculation that the emergence of GBS as a pathogen in southern and eastern Africa is related to HIV infection is tempting. HIV-infected adults have defects in the humoral immune responses to polysaccharide antigens, best recognized in the case of pneumococci (*33*). GBS capsular polysaccharides are similar to pneumococcal capsular polysaccharides, and serologic cross-reactivity is recognized (*34*). Thus, HIV-infected women might carry more GBS and might transfer less transplacental protection. Further research in this area is required.

We found all isolates were susceptible to the β- lactam antimicrobial drugs and that most (96%) were resistant to tetracycline, as would be expected. However, 21% of isolates were resistant to erythromycin, which is a higher proportion than that reported from the United Kingdom (4% erythromycin resistant) (*3*) but similar to that reported from France (21.4%) (*35*), the United States (20%) (*36*), and Zimbabwe (14%) (*37*). Chemoprophylaxis with antenatal azithromycin is under evaluation as a means to improve pregnancy outcome in Malawi, primarily by reducing chorioamnionitis (including that caused by GBS) and possibly malaria. Were this treatment to become available, this higher rate of resistance to macrolides may limit the value of this approach in reducing GBS-associated pathology and could limit options for intrapartum antimicrobial prophylaxis for penicillin-allergic patients.

From our data, interventions to prevent GBS disease appear warranted. Chemoprophylaxis has been successful in reducing rates of EOD in many countries (*2**,**5*). An intrapartum screening–based approach for prophylaxis would not be feasible because microbiology facilities are lacking in both QECH and the surrounding districts. Risk-based prophylaxis could be considered. However, only a small proportion of these infants were of LBW (10), and of these only 7 were noted to be premature. We had insufficient information about the obstetric histories to examine risk factors such as prolonged rupture of membranes, maternal fever, and prolonged labor. Vaginal disinfection with microbicides during labor has been considered in developing countries (*38*). In Malawi, the use of chlorhexidine wipes significantly reduced neonatal and maternal sepsis–related illness and death at QECH in a study in which the primary aim was to reduce perinatal HIV transmission (*39*). This approach is likely to be less effective when a high proportion of deliveries take place without healthcare supervision, and this fact may in part explain the failure of this technique to become routine practice.

A vaccine-based strategy would be particularly suited for use in the developing world, where maternal immunization with tetanus toxoid is a safe and valuable part of routine antenatal care (*40*). However, the impetus to develop these vaccines has diminished because of the success of chemoprophylaxis in industrialized countries. Vaccination would appear to offer the widest coverage for a successful intervention and would likely offer protection from both EOD and LOD. Our study suggests that an efficacious 2-valent vaccine aimed at serotypes Ia and III could prevent >75% of invasive disease due to GBS in Malawian infants.

In summary, we have demonstrated a pattern of neonatal GBS disease similar in scale and serotype distribution to reports from the industrialized world but with a significantly worse outcome. We suggest that the effectiveness of vaginal disinfection should be further assessed and that the currently stalled vaccine development programs of recent years be restarted with a clear intention of assessing their role in the developing world.
